# Haematology of N’Dama and West African Shorthorn cattle herds under natural *Trypanosoma vivax* challenge in Ghana

**DOI:** 10.12688/f1000research.14032.2

**Published:** 2018-08-10

**Authors:** Ebenezer Yaw Ganyo, Johnson N Boampong, Daniel K Masiga, Jandouwe Villinger, Paa Kobina Turkson

**Affiliations:** 1 Department of Biomedical and Forensic Sciences, School of Biological Sciences, University of Cape Coast, Ghana; 2 International Centre of Insect Physiology and Ecology (icipe), Nairobi, Kenya; 3 Department of Animal Science, School of Agriculture, University of Cape Coast, Ghana

**Keywords:** Haematology, cattle, trypanotolerance, trypanosomosis, N’Dama, West African Shorthorn

## Abstract

**Background:** Animal trypanosomosis is a major cause of economic loss in livestock production in Africa. A suggested control measure is to use breeds with traits of trypanotolerance. The study examines the effect of natural *Trypanosoma vivax *challenge on haematological parameters in two trypanotolerant cattle [N’Dama and West African Shorthorn (WASH)] herds.

**Methods:** *Trypanosoma vivax*-specific primers were used to diagnose *T. vivax* infection in an N’Dama herd at Cape Coast in southern Ghana and a WASH herd at Chegbani in northern Ghana from May to July 2011 in a cross-sectional study. Levels of haematological parameters comprising packed cell volume (PCV), haemoglobin (Hb) concentration and red blood cell (RBC) and total white blood cell (TWBC) counts; differential WBC counts (neutrophils, lymphocytes, eosinophils, monocytes and basophils); and RBC indices of mean corpuscular volume (MCV), mean corpuscular haemoglobin (MCH) and mean corpuscular haemoglobin concentration (MCHC) were determined in blood samples and then compared between infected and uninfected cattle.

**Results:** We found that haematological indices for infected and uninfected animals in both breeds were within the normal range. However, the mean PCV values for *T. vivax*-infected WASH and N’Dama were lower in infected compared to uninfected animals. The difference was significant ( *p*< 0.05) in N’Dama but not in WASH.

**Conclusion:** Despite the presence of infection by *T. vivax*, N’Dama and WASH cattle maintained their haematological parameters within acceptable normal ranges,  which confirms their trypanotolerant trait. This highlights the need for low-input traditional African farmers in medium, high and severe tsetse challenge areas to be educated on the advantages of N’Dama and WASH breeds to increase their utilization in integrated tsetse and trypanosomosis control programmes.

## Introduction

Animal trypanosomosis, caused by trypanosomes mainly transmitted by tsetse flies results in annual economic losses in Africa in the range of US$ 1.0 - 1.2 billion in cattle production alone, and more than US$ 4.75 billion in terms of agricultural Gross Domestic Product ( Enyaru *et al.*, 2010). Among species of trypanosomes that cause nagana, *Trypanosoma vivax* is the predominant species in Ghana ( Adam *et al.*, 2012; Mahama *et al.*, 2004; Turkson, 1993) agreeing with reports by Losos (1986) that *T. vivax* predominates in West Africa.

The usual consequence of trypanosome infection is anaemia, which is often accompanied by poor growth, weight loss, low milk yield, infertility, abortion and paralysis ( Berthier *et al.*, 2015; Dagnachew *et al.*, 2015; Mattioli *et al.*, 1999; Steverding, 2008; Trail *et al.*, 1990). Death may result within a few weeks to several months after infection. The use of prophylactic and curative drugs has remained the most popular method for the management of animal trypanosomosis in sub-Saharan Africa (SSA) ( Kinabo, 1993; Mahama *et al.*, 2003; Turkson, 1993). However, the continued use for more than half a century of a limited number of closely related trypanocidal drugs has led to the emergence of drug resistant trypanosomes ( Delespaux *et al.*, 2008; Matovu *et al.*, 2001; Sow *et al.*, 2012). It is unlikely that a vaccine will become available in the foreseeable future ( Nyame *et al.*, 2004; Vale, 2009). Control of tsetse flies, the cyclical vectors of trypanosomes in SSA, is a significant factor in managing the disease in a holistic manner ( Allsopp, 2001; Bouyer *et al.*, 2015; Kgori *et al.*, 2006; Leak *et al.*, 1995; Mulla & Rickman, 1988; Vreysen *et al.*, 2000). Nonetheless, local eradication successes have been limited to less than 2% of the infested area due to either resurgence of residual populations that were omitted from eradication campaigns or reinvasion from neighbouring infested areas ( Bouyer *et al.*, 2015). Landscape friction maps based on tsetse fly genetic distances and remotely sensed environmental data have identified natural barriers to isolated clusters of *Glossina palpalis gambiensis* in West Africa ( Bouyer *et al.*, 2015). This represents a major advance in locating and eradicating isolated tsetse populations without risk of reinvasion. However, this innovation has yet to be replicated at the continental level under the auspices of the Pan African Tsetse and Trypanosomoses Eradication Campaign (PATTEC) ( Bouyer *et al.*, 2015).

The use of trypanotolerant cattle has not been given adequate consideration in integrated tsetse and trypanosomosis control programmes ( Agyemang, 2005; Hendrickx *et al.*, 2004). Trypanotolerant breeds, although equally susceptible to initial infection by trypanosomes, possess the ability to survive, reproduce and remain productive in areas of high tsetse challenge without the need for the use of chemicals to control the vector or drugs to control the parasite ( Dayo *et al.*, 2009; Mattioli *et al.*, 1998; Rege *et al.*, 1994; Trail *et al.*, 1990; Yaro *et al.*, 2016), where other breeds rapidly succumb to the disease ( Berthier *et al.*, 2015; Mattioli *et al.*, 1998; Murray & Dexter, 1988). The trypanotolerant trait is generally attributed to the taurine breeds of cattle in West and Central Africa, namely, the N’Dama and the West African shorthorn (WASH) ( Hoste *et al.*, 1992; Maganga *et al.*, 2017; Mattioli *et al.*, 1998; Mattioli *et al.*, 1999; Roelants, 1986; Trail *et al.*, 1990; Trail *et al.*, 1994). Similar observations have been made for the Orma Boran X Maasai Zebu (Orma Zebu) crossbred cattle in East Africa ( Maichomo *et al.*, 2005; Mwangi *et al.*, 1998a; Mwangi *et al.*, 1998b). Studies have shown that the basis of this trait was associated with the capacity of these animals to develop less severe anaemia in the face of infection ( Berthier *et al.*, 2015; Mattioli *et al.*, 1998; Murray *et al.*, 1982; Murray & Dexter, 1988; Trail *et al.*, 1990).

We previously reported natural *T. vivax* challenge in N’Dama and WASH cattle herds in Ghana using a sensitive PCR approach ( Ganyo, 2014). This study examines the effect of natural *T. vivax* challenge on haematological parameters in these trypanotolerant cattle herds.

## Methods

### Animals, sampling and blood collection

Fifty-five animals each were sampled from an N’Dama herd at Cape Coast in southern Ghana and a WASH herd at Chegbani in northern Ghana from May to July 2011 in a cross-sectional study. Within the same study, 55 animals were sampled from a Sanga herd at Aveyime in the coastal savanna agro-ecological zone and 38 Zebu cattle were sampled in herds at Pong Tamale in the Guinea Savanna agro-ecological zone. The herds were chosen purposively, since these were herds with the breeds of interest. Whereas owners of Sanga and Zebu cattle used trypanocidal drugs regularly to control trypanosome infection, none of the owners of N’Dama and WASH cattle used trypanocidal drugs to control trypanosome infection. From each animal, about 4 ml of blood was collected from the jugular vein using standard operating procedure that required no sedation and transferred into vacutainer tubes containing EDTA as anticoagulant. The vacutainer tubes were then placed in a coolbox containing ice packs for transportation to the laboratory, where they were refrigerated the same day for subsequent analysis.

### Trypanosome detection

DNA was extracted from 200 μl of blood of each animal according to the protocol of Bruford *et al.* (1998) following red blood cell (RBC) lysis ( Biéler *et al.*, 2012). The procedure for DNA amplification and diagnosis of *T. vivax* infection has been described elsewhere ( Ganyo, 2014). Briefly, amplifications were carried out targeting the 170-nucleotide (nt) satellite DNA monomer sequence of *T. vivax*. The PCRs were carried out in 20 μl reaction volumes with 10 pmoles of each primer i.e. TVW_A (5’-GTGCTCCATGTGCCACGTTG-3’) and TVW_B (5’-CATATGGTCTGGGAGCGGGT-3’) ( Masiga *et al.*, 1996), 4.0 μl 5X HF Buffer (Finnzymes), 0.4 μl of 10mM dNTPs, 0.2 μl (1 unit) Taq polymerase (Finnzymes) and 1 μl of DNA template. Cycling conditions for the PCR were accomplished in a 96-well thermocycler (PTC-100 Programmable Thermal Controller, MJ Research, Gaithersburg) as follows: initial denaturation at 98°C for 30 sec, followed by 35 cycles of denaturation at 98°C for 10 sec; annealing at 68°C for 30 sec, primer elongation at 72°C for 15 sec, and a final extension at 72°C for 7 min. PCR products were mixed with loading dye and samples were loaded alongside a molecular weight DNA marker as well as known positives and negatives into 1.5% agarose gel, stained with 0.5ug/ml ethidium bromide. Electrophoresis was set at 75 volts for 1 hr 20 min, followed by visualization of the DNA under UV-illumination.

### Determination of haematological parameters

Packed cell volume (PCV) was determined by the microhaematocrit centrifugation technique while haemoglobin (Hb) concentration was measured spectrophotometrically by the cyanmethaemoglobin method ( Jain, 1986). Red blood cell (RBC) and total white blood cell (TWBC) counts were done manually using a haemocytometer, according to the procedure outlined in Merck Veterinary Manual ( Merck Veterinary Manual, 1986). Differential WBC counts were obtained from air dried thin blood smears stained with Giemsa stain according to the battlement method ( Merck Veterinary Manual, 1986). RBC indices of mean corpuscular volume (MCV), mean corpuscular haemoglobin (MCH) and mean corpuscular haemoglobin concentration (MCHC) were calculated using standard formulae.

### Statistical analysis

The means and standard deviations per breed for haematological parameters for *T. vivax* positive individuals and *T. vivax* negative individuals were calculated using standard formulae.

The non-parametric Kruskal-Wallis test was used to compare the means for haematological parameters in *T. vivax* positive and *T. vivax* negative cattle with the R statistical software version 3.4.2 ( R Development Core Team, 2017). Tests of significance were done at α = 0.05.

## Results

Seven of the N’Dama samples (n=55) and 4 animals from the WASH samples (n=55) were positive for *T. vivax* infection. None of the 55 Sanga and 38 Zebu cattle sampled tested positive for *T. vivax*. The mean haematological values for trypanosome-positive and negative cattle are shown in Table 1. For the N’Dama cattle, significant differences were observed in PCV ( *p* < 0.05), total RBC count, MCV ( *p* < 0.01) and MCH ( *p* < 0.01) values between infected and uninfected cattle, with PCV, MCV and MCH values being significantly higher in uninfected compared to infected cattle. The other parameters were similar for both groups ( Table 1). For the WASH cattle, the PCV, Hb and RBC values for uninfected cattle were higher than those for infected cattle ( Table 1). For the N’Dama cattle, the mean TWBC counts for infected animals were lower than that for uninfected animals. The mean values of neutrophils, lymphocytes and eosinophils were lower in infected N’Dama compared to uninfected N’Dama. The WASH cattle had higher mean TWBC counts for infected animals compared to uninfected animals. The mean values of neutrophils and eosinophils were higher in infected WASH compared with uninfected.

**Table 1.** Within-breed comparison of haematological parameters (mean ± SD) of *Trypanosoma vivax* positive and negative N’Dama cattle at Cape Coast and WASH cattle at Chegbani, Ghana.

	N’Dama (n = 55)			WASH (n = 55)			
Parameter	Positive n = 7	Negative n = 48	*p* value	Positive n = 4	Negative n = 51	*p* value	Reference range ^a^ (mean)
PCV (%)	30.7 ± 4.4	34.6 ± 4.0	0.027 *	28.3 ± 1.5	31.5 ± 5.1	0.167	24.0 - 46.0 (35.0)
Hb (g/dl)	11.8 ± 1.9	13.1 ± 1.6	0.140	11.5 ± 1.1	11.7 ± 2.0	0.746	8.0 - 15.0 (11.0)
RBC (x10 ^6^mm ^3^)	9.1 ± 2.1	6.9 ± 1.9	0.017 *	5.5 ± 1.1	5.8 ± 1.3	0.795	5.0 - 10.0 (7.0)
MCV (fl)	35.8 ± 11.0	53.1 ± 14.1	0.004 **	53.4 ± 12.8	56.6 ± 11.8	0.486	40.0 - 60.0 (52.0)
MCH (pg)	13.8 ± 4.5	20.0 ± 5.2	0.010 **	21.5 ± 4.7	21.0 ± 4.7	0.974	11.0 - 17.0 (14.0)
MCHC (g/dl)	38.6 ± 4.6	38.0 ± 4.4	0.762	40.5 ± 2.0	37.3 ± 4.6	0.086	30.0 - 36.0 (32.7)
TWBC (x10 ^3^mm ^3^)	7.0 ± 2.9	8.6 ± 3.4	0.177	11.5 ± 1.4	10.5 ± 2.2	0.250	4.0 - 12.0 (8.0)
Neutrophils (x10 ^3^mm ^3^)	2.8 ± 2.0	3.5 ± 2.1	0.405	5.3 ± 1.6	4.7 ± 1.5	0.399	0.6 - 5.6 (2.2)
Lymphocytes (x10 ^3^mm ^3^)	2.9 ± 1.5	3.5 ± 1.8	0.614	5.6 ± 2.6	4.5 ± 2.0	0.218	1.8 - 9(4.6)
Eosinophils (x10 ^3^mm ^3^)	1.3 ± 0.4	1.5 ± 0.8	0.479	0.6 ± 0.2	1.0 ± 0.6	0.089	0.0 - 2.4 (0.7)
Monocytes (x10 ^3^mm ^3^)	0.0 ± 0.1	0.0 ± 0.1	0.967	0.0 ± 0.1	0.3 ± 0.4	0.077	0.1 - 0.8 (0.3)
Basophils (x10 ^3^mm ^3^)	0.0 ± 0.0	0.0 ± 0.0	0.586	0.0 ± 0.0	0.0 ± 0.0	0.779	0.0 - 0.2 (0.0)
Neutrophils (%)	38.7 ± 12.8	39.1 ± 13.6	0.940	47.5 ± 19.5	45.0 ± 12.4	0.733	15.0 - 47.0 (28.0)
Lymphocytes (%)	39.6 ± 15.6	40.6 ± 12.0	0.820	47.5 ± 18.5	42.1 ± 13.5	0.322	45.0 - 75.0 (58.0)
Eosinophils (%)	21.4 ± 13.7	20.0 ± 11.5	0.970	4.8 ± 1.7	9.5 ± 5.1	0.042 *	0.0 - 20.0 (9.0)
Monocytes (%)	0.3 ± 0.8	0.2 ± 0.6	0.967	0.3 ± 0.5	2.9 ± 3.5	0.071	2.0 - 7.0 (4.0)
Basophils (%)	0.0 ± 0.0	0.0 ± 0.2	0.586	0.0 ± 0.0	0.0 ± 0.3	0.779	0.0 - 2.0 (0.5)

n represents number of samples in each category *Indicates level of significance at 5% level ( *p*< 0.05) **Indicates level of significance at 1% level ( *p*< 0.01) ^a^ Jain, 1993 PCV, packed cell volume; Hb, haemoglobin; RBC, red blood cells; MCV, mean corpuscular volume; MCH, mean corpuscular haemoglobin; MCHC, mean corpuscular haemoglobin concentration; TWBC, total white blood cells.

Haematological parameters of *T. vivax* infected and uninfected WASH and N´Dama cattle at Chegbani and Cape Coast, respectively Click here for additional data file. Copyright: © 2018 Ganyo EY et al. 2018 Data associated with the article are available under the terms of the Creative Commons Zero "No rights reserved" data waiver (CC0 1.0 Public domain dedication).

## Discussion

The mean PCVs for infected N’Dama and WASH cattle in this study were lower than those for uninfected cattle, which is consistent with the pathological effect of anaemia in trypanosome infections. Our observations support the findings of Berthier *et al.* (2015) that N’Dama and Zebu cattle experimentally infected with *Trypanosoma congolense* had lower PCVs than uninfected animals. Trail *et al.* (1994) also showed that N’Dama naturally infected by trypanosomes in Zaire (now Congo) had lower PCV values and lower weight gain than non-infected N’Dama. A study in Ethiopia ( Dagnachew *et al.*, 2015) in Zebu cattle experimentally infected with *T. vivax* isolates showed that the mean PCV, Hb and total RBC count were lower in infected groups than in non-infected control animals. Additionally, an earlier survey conducted in cattle maintained under traditional management system in 8 villages in Hawagelan district, western Ethiopia ( Bekele & Nasir, 2011) revealed that the mean PCV of trypanosome-infected animals was significantly lower than that of non-infected animals. Lower herd average PCVs for trypanosome-positive cattle compared to trypanosome-negative cattle have also been reported in Ankole cattle in Uganda ( Waiswa & Katunguka-Rwakishaya, 2004), Angoni cattle in Zambia ( Marcotty *et al.*, 2008), Doayo and Zebu White Fulani cattle in Cameroun ( Achukwi & Musongong, 2009) and Zebu cattle in Gabon ( Cossic *et al.*, 2017). In Nigeria, Ohaeri & Eluwa (2011) observed that in addition to cattle, other domestic ruminants that were naturally infected with trypanosomes had significantly lower ( *p* < 0.05) PCV and RBC counts compared to uninfected animals.

The mean PCV, Hb and RBC values observed in both infected and uninfected N’Dama and WASH cattle in current study were within the established normal reference values ( Jain, 1993). The presence of *T. vivax* parasites is associated with a reduction in the mean RBC values below the normal range, an indication of anaemia ( Murray & Dexter, 1988; Silva *et al.*, 1999). However, infected cattle had mean RBC values within the normal range, which could be attributed to their trypanotolerant trait.

With typical trypanotolerance, cattle have the capacity to develop less severe anaemia in the face of pathogenic *Trypanosoma* species infection, as assessed by packed cell volume ( Berthier *et al.*, 2015; Mattioli *et al.*, 1998; Murray *et al.*, 1982; Murray & Dexter, 1988; Trail *et al.*, 1990). Our findings are in agreement with the report ( Mbanasor *et al.*, 2003) that the mean RBC, Hb and PCV values in natural *T. vivax-*infected trypanotolerant Muturu (WASH) cattle in Nigeria were well within accepted normal values for cattle. An earlier study involving trypanotolerant WASH cattle in Ghana also reported normal PCV value despite the presence of trypanosome parasites ( Adam *et al.*, 2012).

In this study, we sampled trypanosusceptible Sanga and Zebu cattle in the same agro-ecological zones as the trypanotolerant N’Dama and WASH. However, none of the trypanosusceptible cattle tested positive for *T. vivax*. Consequently, there was no *T. vivax* positive Sanga and Zebu for which haematological data could be generated for comparison with *T. vivax* positive N’Dama and WASH. Thus, it is not possible to know if the *T. vivax* strains that infected the N’Dama and WASH are highly pathogenic or not. However, such absence of positives is not surprising. Livestock keepers who kept the trypanosusceptible Zebu and Sanga used trypanocidal drugs regularly to control the infection. In contrast, none of the livestock keepers who kept trypanotolerant N’Dama and WASH cattle used trypanocidal drugs to control trypanosome infection. This could explain why some of the N’Dama and WASH tested positive for *T. vivax*, while all the Sanga and Zebu tested negative for *T. vivax*.

Although the importance of keeping trypanotolerant N’Dama and WASH cattle as a trypanosomosis control measure in SSA has long been recognized ( Hoste *et al.*, 1992; Mahama *et al.*, 2003; Pierre, 1906 cited in Agyemang, 2005), these breeds constitute only a small proportion (6%) of the cattle population of Africa and only 17% of the total cattle population in the affected areas ( Agyemang, 2005). The low number of these trypanotolerant animals is attributed in part to the prevalent notion that they are not productive because of their relatively small size. Indeed, in market oriented livestock production systems, livestock keepers by reason of access to cash and consequently to veterinary inputs tend to increase the size of their cattle through crossbreeding trypanotolerant N’Dama and WASH females with trypanosusceptible Zebu bulls. This phenomenon has resulted in the emergence of stabilized crosses such as Sanga in Ghana, Borgou in Togo and Benin, Keteku in Nigeria, Djokoré in Senegal, Bambara in Central African Republic, and Méré in Côte d’Ivoire, Burkina Faso and Mali ( Hendrickx *et al.*, 2004; Hoste *et al.*, 1992). Agyemang (2005) reviewed productivity and economic data from numerous studies (including Agyemang *et al.*, 1991; Agyemang *et al.*, 1997; Lhoste, 1986; Otchere, 1983; Republic of Gambia, 2002; Shaw, 2003; Trail *et al.*, 1979a; Trail *et al.*, 1979b; Wagenaar *et al.*, 1986 and Wilson, 1989) and reiterated the findings that trypanotolerant N’Dama and WASH compare favourably with Zebu in terms of productivity and economic indices even in zero- to low-tsetse challenge areas. Besides the threat that tsetse transmitted trypanosomosis poses to livestock, the productivity of more than one third of the total land area in West Africa is hampered by major physical constraints, the most important being soil fertility ( Hendrickx *et al.*, 2004). Studies indicate that integration of crop and livestock production or mixed crop–livestock farming is the most sustainable means of increasing land productivity in SSA ( McIntire *et al.*, 1992; Winrock, 1992). Accordingly, trypanotolerant cattle have been recommended for low-input traditional African farming systems in areas where tsetse challenge is considered to be medium, high or very severe ( Hendrickx *et al.*, 2004; Mattioli *et al.*, 1998; Shaw & Hoste, 1987; Snow & Rawlings, 1999).

As was clearly demonstrated in the current study, infected trypanotolerant WASH and N’Dama maintained their mean RBC values in the normal range, which confirms their trypanotolerant trait. Further, none of the livestock keepers who kept such trypanotolerant breeds used trypanocidal drugs regularly to control the infection. According to various researchers ( Dayo *et al.*, 2009; Maganga *et al.*, 2017; Mattioli *et al.*, 1998; Rege *et al.*, 1994; Yaro *et al.*, 2016), trypanotolerant breeds possess the ability to survive, reproduce and remain productive in areas of high tsetse challenge without the need for the use of chemicals to control the vector or drugs to control the parasite, despite being equally susceptible to trypanosome infection. This study thus calls attention to the need to deploy trypanotolerant WASH and N’Dama cattle in integrated tsetse and trypanosomosis control programmes in low-input traditional African farming systems in medium, high and severe tsetse challenge areas. A key component of such programs should be education of farmers on advantages of N’Dama and WASH cattle to establish and rapidly increase their numbers.

The mean values of TWBC, neutrophil and lymphocyte counts for *T. vivax* infected N’Dama in the present study were lower than those for the uninfected N’Dama. Authie (1993) observed that leukopenia is induced by trypanosomosis. Silva *et al.* (1999) reported that the main haematological changes produced by *T. vivax* infections in cattle in the Bolivian Wetlands and Brazilian Pantanal were anaemia and severe leucopenia. Additionally, the natural occurrence of *T. vivax* in cattle in Mosul, Iraq, resulted in leucopenia due to lymphopenia and neutropenia in comparison with normal range for cattle ( Rhaymah & Al-Badrani, 2012). Paling *et al.* (1991) and Berthier *et al.* (2015), however, observed leukocytosis in N’Dama cattle due to trypanosome infection. The mean TWBC, neutrophil and lymphocyte counts for *T. vivax* infected WASH cattle in this study were higher than those for the uninfected. Similar observations of leukocytosis due to trypanosome infection have been made in rabbits ( Emeribe & Anosa, 1991), vervet monkeys ( Kagira *et al.*, 2006) and Zebu cattle ( Berthier *et al.*, 2015; Van Wyk *et al.*, 2014). Ilemobade *et al.* (1982) suggested that trypanosome-induced severe leukopenia could compromise the protective immunity of cattle to other diseases. High levels of neutrophils, lymphocytes and eosinophils may indicate an active infection, whereas low counts may indicate a compromised immune system ( Davidson *et al.*, 1998; Spivak, 1984). The mean TWBC counts reported in this study were within the normal values for cattle ( Jain, 1993), but were lower than those reported in both *T. vivax* infected and uninfected Muturu cattle in Nigeria ( Mbanasor *et al.*, 2003), which were higher than the normal values for cattle. The differential WBC counts in this study were within the normal range ( Jain, 1993) and agree with what has been reported by Mbanasor *et al.* (2003).

## Conclusion

We found that in spite of the presence of natural *T. vivax* infection, the haematological parameters of N’Dama and WASH cattle were within acceptable normal ranges, which confirms their trypanotolerant trait. This underscores the need for low-input traditional African farmers in medium, high and severe tsetse challenge areas to be educated on their advantages in order to establish and rapidly increase their numbers for effective deployment in integrated tsetse and trypanosomosis control programmes.

### Statement of animal welfare and ethics

Collection of blood was done as per standard operating procedures to ensure animal welfare ( https://www.dpi.nsw.gov.au/animals-and-livestock/animal-welfare/general/general-welfare-of-livestock/sop/pigs/health/blood-collection). These are standard operating procedures used in veterinary medicine internationally.

Owners of animals gave their consent before the animals were bled. Prior to jugular venipuncture, the body of the animal was manually restrained by assistants to avoid injury to the animal. Further, the head of the animal was turned by another assistant at a 30-degree angle to the side by holding the animal under its jaw; this is to allow for easy access to the vein, and, to ensure quick, easy and safe collection of the sample causing minimal distress to the animal. To avoid repeated puncturing, time was taken to locate the vein accurately and it was distended by gentle pressure with the fingers before the needle was inserted. After the vein was located, the area was properly cleaned by alcohol to keep bacteria out of the needle insertion site. To ensure that sampling did not result in hypovolemic shock, physiological stress, anaemia and possibly death, only a minimal amount of 4ml of blood was drawn from each animal. To prevent needle-stick injury, a new needle was used for each venipuncture. As soon as blood was removed from the animal, the insertion site was swabbed with alcohol to remove any bacteria that might have entered the area during the drawing of blood. Pressure was applied for 30–60 seconds immediately following withdrawal of the needle; the pressure caused blood to clot, thereby preventing bleeding.

At the time this work was conducted (2011) there was no requirement by the University of Cape Coast for ethical clearance for work with animals. Therefore, we followed internationally accepted procedures such as those outlined in "Guidelines for the Welfare of Livestock from which Blood is Harvested for Commercial and Research Purposes” published by the New Zealand National Animal Ethics Advisory Committee in 2009 ( https://www.mpi.govt.nz/dmsdocument/1475-guidelines-for-the-welfare-of-livestock-from-which-blood-is-harvested-for-commercial-and-research-purposes).

## Data availability

The data referenced by this article are under copyright with the following copyright statement: Copyright: © 2018 Ganyo EY et al.

Data associated with the article are available under the terms of the Creative Commons Zero "No rights reserved" data waiver (CC0 1.0 Public domain dedication). 

Dataset 1: Haematological parameters of *T. vivax* infected and uninfected WASH and N'Dama cattle at Chegbani and Cape Coast, respectively. 10.5256/f1000research.14032.d213201 ( Ganyo *et al.*, 2018).

## Author information

EYG holds a PhD in Parasitology. JNB is an Associate Professor in the Department of Biomedical and Forensic Sciences, and the Dean of the School of Biological Sciences. JV is a scientist and head of the Molecular Biology and Bioinformatics Unit, International Centre of Insect Physiology and Ecology Nairobi, Kenya. DKM is the head of Animal Health, International Centre of Insect Physiology and Ecology, Nairobi, Kenya. PKT is a Professor of Veterinary Epidemiology and Dean, School of Veterinary Medicine, University of Ghana, Legon, Accra, Ghana.
