# Supplementary material for: Haematology of N’Dama and West African Shorthorn cattle herds under natural Trypanosoma vivax challenge in Ghana
Source: F1000Res. 2018 Aug 10;7:314. Originally published 2018 Mar 13. [Version 2] doi: 10.12688/f1000research.14032.2 (PMC6117849; doi:10.12688/f1000research.14032.2)
Supplement: Peer review history

# Peer review: Referee response for version 2

**DOI:** 10.5256/f1000research.17131.r36986

**Published:** 2018-09-26

**Authors:** David M Groth

**Affiliations:** 1 School of Biomedical Sciences, Curtin University, Bentley, WA, Australia

This version is acceptable for indexing.

I have read this submission. I believe that I have an appropriate level of expertise to confirm that it is of an acceptable scientific standard.

# Peer review: Referee response for version 2

**DOI:** 10.5256/f1000research.17131.r36985

**Published:** 2018-08-29

**Authors:** Sophie Thévenon

**Affiliations:** 1 UMR INTERTRYP, French Agricultural Research Centre for International Development (CIRAD) , Montpellier, France

The manuscript has been improved.

 Some terms must still be corrected: “infected” and “uninfected” must not be used in the manuscript and must be replaced everywhere by T. vivax PCR-negative and T vivax PCR-positive

 Results and discussion section: there is not any significant differences between positive and negative animals, concerning blood formula, thus the figures must not be over-interpreted and they should not be discussed so widely.

I have read this submission. I believe that I have an appropriate level of expertise to confirm that it is of an acceptable scientific standard, however I have significant reservations, as outlined above.

# Peer review: Referee response for version 1

**DOI:** 10.5256/f1000research.15253.r33786

**Published:** 2018-06-01

**Authors:** Sophie Thévenon

**Affiliations:** 1 UMR INTERTRYP, French Agricultural Research Centre for International Development (CIRAD) , Montpellier, France

The manuscript aims at assessing the effect of infections by Trypanosoma vivax on the hematological parameters of N’dama and WASH (West African Short-horn) cattle, raised in two natural environments in Ghana. The purpose is to highlight the trypanotolerant character of these breeds.

 Major comments:

 The article suffers from several major problems and is not suited for indexing.

 The bibliography is quite incomplete: major papers written by Trail et al ^1^^-^^2^ and Mattioli et al ^3^^-^^4^ are not cited. These authors worked on N’Dama cattle raised in Congo and Gambia respectively and on the relationships between productivity, anemia and infections. Mattioli et al 1998 (Acta Tropica) showed that N’Dama cattle suffered from high tse-tse challenge. Trail et al 1994 showed that N’dama infected by trypanosomes had lower PCV values and lower weight gain than non-infected N’Dama. In addition, an experimental infection published by Berthier et al (2015) ^5^, presented anemia evolution in 5 cattle breeds of West Africa under T. congolense infection and show of N’Dama and WASH were less anemiated than Zebu Fulani and Borgou.

 The experimental design presented in the article does not bring robust elements on anemia control during T. vivax infection and on the comparison between N’Dama and WASH. There is not any susceptible breed that could be compared to N’Dama and WASH. It is thus not possible to know if the T. vivax strains are highly pathogenic or not. Since N’Dama and WASH are not raised in the same area under the same agro-ecological context, it is not possible to compare these two breeds.

 Because only 4 and 7 animals were positive to T. vivax PCR, an Anova cannot be used. Only a non-parametric test can be used.

 Other comments:

 The article of Bouyer et al (2015) ^6^ must be cited in the introduction concerning control method. I do not agree with the sentence “past and current control methods are limited”: the use of trypanocide drugs may be useful and efficient when their usage is adapted to the context (environment and breeding system).

 In the table I and II and in the text, the terms “positive in T. vivax PCR” and “negative in T. vivax PCR” must be used instead of infected or uninfected. Indeed, PCR has a sensitivity around 75-80% and thus some animals considered as negative in PCR may be infected.

 In the discussion, the authors propose to incorporate routine diagnosis and treatment. But the problem is that there is not any routine diagnosis, since parasitological methods have a very low sensitivity, and PCR and serology require a well-equipped laboratory with well-trained technicians. Farmers need the support of farmer’s organization and from veterinary public service. The notion of “reservoirs” due to trypanotolerant cattle has never been clearly investigated.

 Finally, the raise of trypanotolerant breeds is important in some agro-ecological context, where tsetse challenge is high and in low input systems. In some areas, only trypanotolerant breed can survive.

I have read this submission. I believe that I have an appropriate level of expertise to state that I do not consider it to be of an acceptable scientific standard, for reasons outlined above.

## References

[1] TrailJCd'IeterenGDFeronAKakieseOMulungoMPeloM: Effect of trypanosome infection, control of parasitaemia and control of anaemia development on productivity of N'Dama cattle. *Acta Trop*.1990;48(1) :37-45 198080210.1016/0001-706x(90)90063-6

[2] TrailJCWissocqNd'IeterenGDKakieseOMurrayM: Quantitative phenotyping of N'Dama cattle for aspects of trypanotolerance under field tsetse challenge. *Vet Parasitol*.1994;55(3) : 10.1016/0304-4017(94)00649-W 185-95 10.1016/0304-4017(94)00649-W 7879377

[3] MattioliRCFayeJABüscherP: Susceptibility of N'Dama cattle to experimental challenge and cross-species superchallenges with bloodstream forms of Trypanosoma congolense and T. vivax. *Vet Parasitol*.1999;86(2) :83-94 1049669210.1016/s0304-4017(99)00117-x

[4] MattioliRJaitnerJCliffordDPandeyVVerhulstA: Trypanosome infections and tick infestations: susceptibility in N’Dama, Gobra zebu and Gobra×N’Dama crossbred cattle exposed to natural challenge and maintained under high and low surveillance of trypanosome infections. *Acta Tropica*.1998;71(1) : 10.1016/S0001-706X(98)00051-5 57-71 10.1016/S0001-706X(98)00051-5 9776143

[5] BerthierDPeylhardMDayoGKFloriLSyllaSBollySSakandeHChantalIThevenonS: A comparison of phenotypic traits related to trypanotolerance in five west african cattle breeds highlights the value of shorthorn taurine breeds. *PLoS One*.2015;10(5) : 10.1371/journal.pone.0126498 e0126498 10.1371/journal.pone.0126498 25954819PMC4425517

[6] BouyerJDickoAHCecchiGRavelSGuerriniLSolanoPVreysenMJDe MeeûsTLancelotR: Mapping landscape friction to locate isolated tsetse populations that are candidates for elimination. *Proc Natl Acad Sci U S A*.2015;112(47) : 10.1073/pnas.1516778112 14575-80 10.1073/pnas.1516778112 26553973PMC4664298

# Peer review: Referee response for version 1

**DOI:** 10.5256/f1000research.15253.r33583

**Published:** 2018-05-18

**Authors:** David M Groth

**Affiliations:** 1 School of Biomedical Sciences, Curtin University, Bentley, WA, Australia

Animal trypanosomosis is an important disease in both animals and humans and understanding the host’s response to such infection is important scientific endeavor. The manuscript describes some basic hematological parameters in these two breeds of animals, which may have some biological significance and lead to some understanding of parameters affecting disease resistance. However, the number of infected animals is quite low in both groups, with only 4/55 WASH and 7/55 N’Dama infected.

 The data supports the conclusions presented.

 Suggestions:

 The use of real numbers for each WB cell subgroup parameter rather than reporting only a %. For instance the Eosinophils in Table 2 could be represented as a number rather than a % and this would give a greater feel for the level of absolute differences. If for instance the Eosinophils were represented as a number then the differences between the breeds would be  much clearer. Both absolute numbers and % could be used.

 5mg/ul is a considerable concentration of ethidium bromide (needs to be checked). Green and Sambrook recommends use of 0.5ug/ml suggest checking the value.

 Concentration of the template is not given and should be.

 Suggest checking concentrations of the reagents used in the PCR. For instance, 10mM dNTPs is quite a considerable quantity of dNTPs or is it x ul of a 10mM dNTP solution. Typical final concentrations in PCR are between 200-250uM.

I have read this submission. I believe that I have an appropriate level of expertise to confirm that it is of an acceptable scientific standard, however I have significant reservations, as outlined above.

# Peer review: Referee response for version 1

**DOI:** 10.5256/f1000research.15253.r33102

**Published:** 2018-04-25

**Authors:** Yahaya Adam

**Affiliations:** 1 Tsetse and Trypanosomiasis Control Unit/PATTEC, Ministry of Food and Agriculture, Pong-Tamale, Ghana

Animal trypanosomosis, as reported in the paper, is indeed a major constraint to livestock production systems in Ghana. The theme for the paper is therefore very appropriate. The study design and methods as presented appear to satisfy the standard requirements for a scientific study. The authors suggested the use of these trypano-tolerant animals as control measure for the problem of animal trypanosomosis but I hold a dissenting view to that. The N'dama and WASH cattle, even though are trypano-tolerant as indicated clearly in the study, can not be a solution to the problem for the following reasons:

 1) The T. vivax  challenge does not mean a 100% free from the impact as demonstrated in the study (PCV of infected slightly lower than that of uninfected for both breeds of the trypano-tolerant animals used in the study).

 2) The N'dama and the WASH breeds are not very productive compared to the other breeds of cattle raised in Ghana, probably due to the T. vivax challenge.

 3) The two breads of cattle in the study have the potential status as reservoir of trypanosomes to other breeds of cattle.

I have read this submission. I believe that I have an appropriate level of expertise to confirm that it is of an acceptable scientific standard.

# Response

**Published:** 2018-04-25

**Authors:** Paa Kobina

We accept the comments of the Referee. We agree that the two breeds are not as productive as other breeds but having them deal better with trypanosomosis is considered by some livestock keepers as an advantage to keep them and therefore may be preferred..
